# Health impact of delayed implementation of cervical cancer screening programs in India: A modeling analysis

**DOI:** 10.1002/ijc.31823

**Published:** 2018-10-16

**Authors:** Nicole G. Campos, Vivien Tsu, Jose Jeronimo, Catherine Regan, Stephen Resch, Andrew Clark, Stephen Sy, Jane J. Kim

**Affiliations:** ^1^ Center for Health Decision Science Harvard T.H. Chan School of Public Health Boston MA; ^2^ PATH, Reproductive Health Global Program Washington DC; ^3^ Global Coalition against Cervical Cancer Arligton VA; ^4^ Department of Health Services Research and Policy London School of Hygiene & Tropical Medicine London United Kingdom

**Keywords:** cervical cancer, screening, HPV DNA testing, VIA, mathematical modeling

## Abstract

India has the highest burden of cervical cancer in the world. To estimate the consequences of delaying implementation of organized cervical cancer screening, we projected the avertable burden of disease under different implementation scenarios of a screening program. We used an individual‐based microsimulation model of human papillomavirus (HPV) infection and cervical cancer calibrated to epidemiologic data from India to project age‐specific cancer incidence and mortality reductions associated with screening (once‐in‐a‐lifetime among women aged 30–34 years) with one‐visit visual inspection with acetic acid (VIA) and one‐ and two‐visit HPV DNA testing. We then applied these reductions to a population model to project the lifetime cervical cancer cases and deaths averted under different implementation scenarios taking place from 2017 to 2026: (1) immediate implementation of screening with currently available screening tests (one‐visit VIA, two‐visit HPV testing); (2) immediate implementation of screening with currently available screening tests, with a switch to point‐of‐care one‐visit HPV testing in 5 years; and (3) 5‐year delayed implementation of screening with current screening tests or point‐of‐care HPV testing. Immediate implementation of two‐visit HPV testing with a switch to one‐visit HPV testing averted 574,100 cases and 382,500 deaths over the lifetimes of 81.4 million 30‐ to 34‐year‐old women screened once between 2017 and 2026. Delayed implementation with a one‐visit HPV test averted 209,300 cases and 139,100 deaths. Delaying implementation of screening programs in high‐burden settings will result in substantial morbidity and mortality among women beyond the age for adolescent HPV vaccination.

## Abstract 2

**What's new?**

Nearly one‐quarter of cervical cancer cases worldwide occur in India. Nonetheless, while the disease can be prevented through screening for precancerous lesions, very few Indian women receive Pap tests. Here, the authors estimated cervical cancer burden in India assuming different screening program implementation scenarios, including immediate implementation with both one‐visit VIA and two‐visit human papillomavirus (HPV) testing and delayed implementation with a one‐visit HPV test. Models showed that immediate implementation of two‐visit HPV testing averted more than double the number of cases and deaths from cervical cancer among 30‐ to 34‐year‐old women compared with delayed implementation with one‐visit HPV testing.

**Abbreviations**
CIN: cervical intraepithelial neoplasia
GDP: gross domestic product
HPV: human papillomavirus
VIA: visual inspection with acetic acid
WHO: World Health Organization.

## Background

India has the largest burden of cervical cancer in the world, with an estimated 123 000 incident cases occurring annually.^1^ This accounts for approximately 23% of cases worldwide, and 32% of cases in less developed countries.^1^ Yet the disease is preventable through either prophylactic vaccination against human papillomavirus (HPV)—the sexually transmitted virus that causes cervical cancer—or screening and treatment of precancerous lesions caused by persistent HPV infection. While the introduction and scale‐up of adolescent HPV vaccination programs would substantially reduce the number of cervical cancer cases in years to come, the full benefits of HPV vaccination will not be realized for more than 30 years; to date, there are several HPV vaccination demonstration projects in India, but the vaccine is not available through the national immunization program.^2^ In the interim, screening is the only form of prevention for the two to three generations of women beyond the target age of adolescent vaccination. Only an estimated 3.1% of women in India reported receiving a Pap smear in the last 3 years.^3^

The World Health Organization (WHO) recommends screening with HPV DNA testing where resources are available; visual inspection with acetic acid (VIA) represents an acceptable alternative in low‐resource settings.^4^ Recent guidelines from the American Society for Clinical Oncology recommend HPV testing for all resource levels, and if resources are not available, VIA should be offered with the goal of developing screening infrastructure and switching to HPV testing as soon as possible.^5^ Promising developments to improve the delivery of preventive care in low‐resource settings include the introduction of low‐cost HPV tests with samples collected by either a health provider or the woman herself;^6^ advances in the use of mobile phones for reaching patients;^7^ and introduction of preventive treatment technologies that do not rely on compressed gas, which may be difficult and costly to obtain.^8^ Additionally, new clinically validated technology exists to facilitate point‐of‐care (i.e., one‐visit screen‐and‐treat) HPV testing, but has not yet been implemented outside of small demonstration projects.^9^ But like other lower‐middle income settings, India faces shortages of health workers, insufficient referral processes and financial constraints that have impeded the success of organized screening that systematically targets eligible women. While operational guidelines developed by the Ministry of Health recommend screening with VIA every 5 years for women over age 30 years, with initial implementation in 100 districts providing data, states will need to fund and implement broader efforts.^10^, ^11^ Without substantial political will and the injection of government and donor funds, the implementation of screening programs will likely continue to stall.

To provide information on the consequences of further delays in the implementation of cervical cancer screening, we quantified the potential health impact of an organized national screening program in India by estimating the cumulative number of cervical cancer cases and deaths averted over the lifetimes of women aged 30–34 years under different implementation scenarios taking place from 2017 to 2026: (1) immediate implementation of screening with currently available screening tests (i.e., VIA, HPV DNA testing); (2) immediate implementation of screening with currently available screening tests, with a switch to point‐of‐care HPV testing in 5 years; and (3) five‐year delayed implementation of screening with current screening tests or point‐of‐care HPV testing.

## Methods

### Study design and data sources

We used an individual‐based microsimulation model of the natural history of HPV infection and cervical cancer^12^—calibrated to epidemiologic data on cervical disease burden in India^13^—to estimate the reductions in cervical cancer incidence and mortality attributable to screening with either VIA or HPV DNA testing. Assumptions and data inputs pertaining to the effectiveness of each test, including coverage of the target population, test performance, compliance with recommended follow‐up, eligibility for cryotherapy and treatment efficacy, are presented in Table 1.^1^, ^6^, ^13^, ^14^, ^15^, ^16^, ^17^, ^18^, ^19^ We then applied the percent reductions in cervical cancer incidence and mortality in each five‐year age group from ages 30 to 79 years to population and disease burden data using the Excel‐based CERVIVAC model, a tool developed for the Pan American Health Organization's (PAHO) ProVac Initiative that contains separate modules for evaluating the costs and health impact associated with either HPV vaccination or cervical cancer screening and treatment of precancerous lesions. Female population data by birth cohort were based on estimates for India through 2100 from the United Nations Population Division (the 2012 revision).^17^ Cervical cancer incidence and mortality rates in the absence of screening were based on GLOBOCAN 2012, produced by the International Agency for Research on Cancer (IARC).^1^ The CERVIVAC model was used to estimate the numbers of cervical cancer cases and deaths averted for each implementation scenario based on screening test, age at screening, screening coverage of the target population and year of implementation.

**Table 1:** Values and data sources for model variables

Variable	Value	Data source
Age of target population	30–34 years	Assumption
**Population coverage of screening program**		
Immediate implementation (2017)	10% (2017–2021), 20% (2022–2026)	Assumption
Delayed implementation (2022)	10% (2022–2026)	Assumption
**Individual‐based Monte Carlo simulation model**		
Loss to follow‐up per visit^a^	20%	Assumption
Test sensitivity/specificity for CIN2+		
VIA	0·55/0·92	Jeronimo *et al*. 2014^6^
HPV DNA test (self‐collection)^b^	0·76/0·95	
**Eligibility for cryotherapy** c		
No lesion or CIN1	100%	Campos *et al*. 2015^14^
CIN2	85%	
CIN3	75%	
Cancer	10%	
Effectiveness of cryotherapy to treat CIN2/3	92%	Sauvaget *et al*. 2013^15^
		Chirenje *et al*. 2001^16^
Test sensitivity/specificity for CIN1+, colposcopy^d^	0·50/0·96	Jeronimo *et al*. 2014^6^
Effectiveness of cryotherapy/LEEP following colposcopy to treat CIN2/3	96%	Chirenje *et al*. 2001^16^
**CERVIVAC population model**		
Population and demographic data^e^	By age and year	United Nations World Population Prospects, 2012 Revision 17
Cervical cancer incidence	By age, in 5‐year groups^f^	Globocan 2012^1^
Cervical cancer mortality	By age, in 5‐year groups^f^	Globocan 2012^1^
Reduction in cervical cancer incidence attributable to screening	By age, in 5‐year groups	Individual‐based Monte Carlo microsimulation model^13^ calibrated to (1) age‐specific prevalence of high‐risk HPV in Hyderabad^6^ and (2) the Nagpur cervical cancer registry (1998–2002)^18^
Reduction in cervical cancer mortality attributable to screening	By age, in 5‐year groups	Individual‐based Monte Carlo microsimulation model^13^ calibrated to (1) age‐specific prevalence of high‐risk HPV in Hyderabad^6^ and (2) the Nagpur cervical cancer registry (1998–2002)^18^

CIN: cervical intraepithelial neoplasia; HPV: human papillomavirus; LEEP: loop electrosurgical excision procedure; VIA: visual inspection with acetic acid.

a Loss to follow‐up was defined as the proportion of women who do not return for each subsequent clinical encounter, relative to the previous visit. In the two‐visit HPV DNA test strategy, loss to follow‐up applied to the results/cryotherapy visit. In the one‐visit VIA, two‐visit HPV DNA test, and one‐visit HPV DNA test strategies, loss to follow‐up applied to both the diagnostic confirmation and treatment visits for women who were not eligible for cryotherapy immediately following a positive screening test.

b HPV DNA test performance characteristics (for self‐collection of vaginal samples) were based on a demonstration project of the *care*HPV test in Hyderabad. We assumed the same test performance characteristics for one‐ and two‐visit HPV testing strategies.

c Eligibility for cryotherapy immediately following a positive screening test was based upon a woman's true underlying health state in the model.

d Test performance characteristics of colposcopy in the PATH START‐UP demonstration project were derived from the worst diagnosis of the local pathologist relative to the worst diagnosis by a quality control pathologist (gold standard); we applied the treatment threshold of CIN1+, although this was not the treatment threshold in the START‐UP project. To derive test performance of colposcopy, we excluded histological classifications that were inadequate or with a histological classification other than negative, CIN1, CIN2, CIN3, or cancer. Because CIN1 is not a true underlying health state in the microsimulation model, performance of colposcopy in the model is based on the underlying health states of no lesion, HPV infection, CIN2, or CIN3. For a treatment threshold of CIN1, we weighted sensitivity of colposcopy for women with HPV based on the prevalence of CIN1 among women with HPV infections in the START‐UP studies.

e Data from the United Nations World Population Prospects^17^ project female population sizes for single ages and single years from 1950 until 2100.

f Globocan 2012^1^ data was provided by the Institut Catala d'Oncologia for a previously published analysis^19^ in 5‐year age groups from ages 30 to 49 years. Globocan data is collapsed into one age group for cervical cancer incidence and mortality rates over 75 years; in the CERVIVAC model, we linearly interpolated the rates in the oldest age groups, assuming that the Globocan data for 75 years and over applied to women aged 85–89 years and then decreased at a linear rate.

### Statistical analysis

The individual‐based microsimulation model of the natural history of HPV infection and cervical cancer, as well as the model calibration process, have been previously described.^12^, ^13^ In brief, the model tracks individual girls who enter the model at age 9 years with a healthy cervix and then transition between mutually exclusive health states that include HPV infection, cervical intraepithelial neoplasia (CIN) (grade 2 or 3) and invasive cervical cancer. As individuals age, they can acquire HPV infections, which may either clear or progress to CIN2 or CIN3. Women with CIN2 or CIN3 may regress or progress to invasive cancer, which can be detected at the local, regional, or distant stage. Transition probabilities may vary by age, HPV type (stratified by HPV 16, 18, 31, 33, 45, 52, 58, other high‐risk types and low‐risk types), duration of infection or CIN status and naturally acquired immunity from prior HPV infection. Death from noncervical causes can occur in any health state, depending on a woman's age, and stage‐specific excess mortality due to cervical cancer can occur after its onset.

To calibrate the model to epidemiologic data on age‐specific high‐risk HPV prevalence^6^ and cervical cancer incidence from India,^18^ we estimated baseline “prior” input parameter values for natural history transitions using longitudinal data.^20^, ^21^, ^22^, ^23^ To reflect heterogeneity in age‐ and type‐specific HPV incidence between India and input source study locations, as well as uncertainty surrounding natural immunity following initial infection and progression and regression of CIN2 and CIN3, we set plausible ranges around these input parameter values. Repeated model simulations (in the absence of any intervention) selected a single random value from the plausible range for each uncertain parameter, creating a unique natural history input parameter set. For each unique parameter set, we computed a goodness‐of‐fit score by summing the log‐likelihood of model‐projected outcomes to represent the quality of fit to epidemiologic data from India (i.e., calibration targets). We selected the top 50 good‐fitting input parameter sets to use in analyses as a form of probabilistic sensitivity analysis. Natural history model inputs for the India microsimulation model, as well as model fit to epidemiologic data, are presented in the Appendix.

We used the calibrated microsimulation model to estimate the average percent reduction in cervical cancer incidence and mortality in each five‐year age group (from ages 30 to 79 years; reductions in women aged 80 to 100 years were assumed to be the same as in women aged 75–79 years) associated with the following screening tests, administered once in a lifetime, relative to no intervention: (1) one‐visit VIA; (2) two‐visit HPV DNA testing; and (3) one‐visit point‐of‐care HPV DNA testing (Table 2) at screening coverage levels of 10% and 20% of women aged 30–34 years. For VIA, we assumed that women who were screen‐positive and eligible for cryotherapy were treated at the same clinical visit. For two‐visit HPV DNA testing, we assumed women self‐collected a vaginal HPV specimen during an initial clinical encounter, and subsequently received results and treatment (if HPV‐positive and eligible for cryotherapy) in a separate visit. For one‐visit HPV DNA testing, we assumed that self‐collection and treatment of HPV‐positive eligible women took place in a single visit. In all strategies, women who were not eligible for cryotherapy were referred to further diagnostic testing with colposcopy, and subsequent treatment if they received a histologic diagnosis of CIN1 or higher. Loss to follow‐up rates were assumed to be 20% per clinical encounter.

**Table 2:** Screening strategies and scale‐up scenarios [Color table can be viewed at wileyonlinelibrary.com]

Strategy	Coverage level (% of women aged 30–34 years) and screening test by year									
	2017	2018	2019	2020	2021	2022	2023	2024	2025	2026
Immediate implementation of currently available tests										
Immediate VIA	10	10	10	10	10	20	20	20	20	20
	1‐v VIA	1‐v VIA	1‐v VIA	1‐v VIA	1‐v VIA	1‐v VIA	1‐v VIA	1‐v VIA	1‐v VIA	1‐v VIA
Immediate 2‐visit HPV	10	10	10	10	10	20	20	20	20	20
	2‐v HPV	2‐v HPV	2‐v HPV	2‐v HPV	2‐v HPV	2‐v HPV	2‐v HPV	2‐v HPV	2‐v HPV	2‐v HPV
Number of women screened	5,185,700	5,252,500	5,309,800	5,359,100	5,399,000	10,862,300	10,916,600	10,969,200	11,025,800	11,096,500
Immediate implementation of currently available tests followed by a switch to point‐of‐care HPV testing										
Immediate VIA, switch to 1‐visit HPV	10	10	10	10	10	20	20	20	20	20
	1‐v VIA	1‐v VIA	1‐v VIA	1‐v VIA	1‐v VIA	1‐v HPV	1‐v HPV	1‐v HPV	1‐v HPV	1‐v HPV
Immediate 2‐visit HPV, switch to 1‐visit HPV	10	10	10	10	10	20	20	20	20	20
	2‐v HPV	2‐v HPV	2‐v HPV	2‐v HPV	2‐v HPV	1‐v HPV	1‐v HPV	1‐v HPV	1‐v HPV	1‐v HPV
Number of women screened	5,185,700	5,252,500	5,309,800	5,359,100	5,399,000	10,862,300	10,916,600	10,969,200	11,025,800	11,096,500
Delayed implementation of currently available tests or point‐of‐care HPV testing										
Delayed VIA	–	–	–	–	–	10	10	10	10	10
						1‐v VIA	1‐v VIA	1‐v VIA	1‐v VIA	1‐v VIA
Delayed 2‐visit HPV	–	–	–	–	–	10	10	10	10	10
						2‐v HPV	2‐v HPV	2‐v HPV	2‐v HPV	2‐v HPV
Delayed 1‐visit HPV	–	–	–	–	–	10	10	10	10	10
						1‐v HPV	1‐v HPV	1‐v HPV	1‐v HPV	1‐v HPV
Number of women screened	–	–	–	–	–	5,431,200	5,458,300	5,484,600	5,512,900	5,548,200

1‐v: 1‐visit strategy; 2‐v: 2‐visit strategy; HPV: HPV DNA testing; VIA: visual inspection with acetic acid. VIA testing is indicated by blue squares (light blue: 10% coverage; dark blue: 20% coverage). 2‐visit HPV testing is indicated by the lavender shading (light lavender: 10% coverage; dark lavender: 20% coverage). 1‐visit (point‐of‐care) HPV testing is indicated by the yellow shading (light yellow: 10% coverage; dark yellow: 20% coverage). Each year, the relevant coverage level was applied to each birth cohort of women between age 30 and 34 years. Thus, depending on the year, cohort, and coverage level, effective coverage for a particular birth cohort could be zero (e.g., for 34 year old women in 2017 under delayed implementation scenarios), or nearly 100% (e.g., for 30 year old women in 2022 after immediate implementation with a switch to 1‐visit HPV testing).

The CERVIVAC model was then used to estimate the number of cervical cancer cases and deaths averted over the lifetimes of women aged 30–34 years for the following implementation scenarios between 2017 and 2026, relative to no screening: (1) immediate implementation of screening with one‐visit VIA beginning in 2017 (“Immediate VIA”); (2) immediate implementation of screening with two‐visit HPV DNA testing beginning in 2017 (“Immediate two‐visit HPV”); (3) immediate implementation of screening with VIA beginning in 2017 and switching to point‐of‐care HPV testing (i.e., one‐visit HPV testing) in 2022 (“Immediate VIA, switch to one‐visit HPV”); (4) immediate implementation of screening with two‐visit HPV DNA testing beginning in 2017 and switching to one‐visit HPV testing in 2022 (“Immediate two‐visit HPV, switch to one‐visit HPV”); (5) delayed implementation of screening with one‐visit VIA, beginning in 2022 (“Delayed VIA”); (6) delayed implementation of screening with two‐visit HPV DNA testing, beginning in 2022 (“Delayed two‐visit HPV”); (7) delayed implementation of screening with one‐visit point‐of‐care HPV testing in 2022 (“Delayed one‐visit HPV”). Screening modalities were selected based on India's operational guidelines, which recommend VIA,^11^ and HPV DNA testing, which has been demonstrated as cost‐effective in previous modeling work.^13^ For strategies involving immediate implementation, we assumed initial coverage was 10% each year of each birth cohort of women aged 30–34 years in that year for the first 5 years of the screening program (2017–2021), and then increased to 20% each year of each birth cohort aged 30–34 years in the next 5 years of the program (2022–2026). For strategies involving delayed implementation, we assumed no screening from 2017 to 2021 and 10% coverage each year of each birth cohort of women aged 30–34 years from 2022 to 2026. Thus, depending on the year, cohort and coverage level, effective coverage for a particular birth cohort considered could be zero (e.g., for 34 year old women in 2017 under delayed implementation scenarios), or nearly 100% (e.g., for 30 year old women in 2022 after immediate implementation with a switch to 1‐visit HPV).

### Results

**Figure 1:**
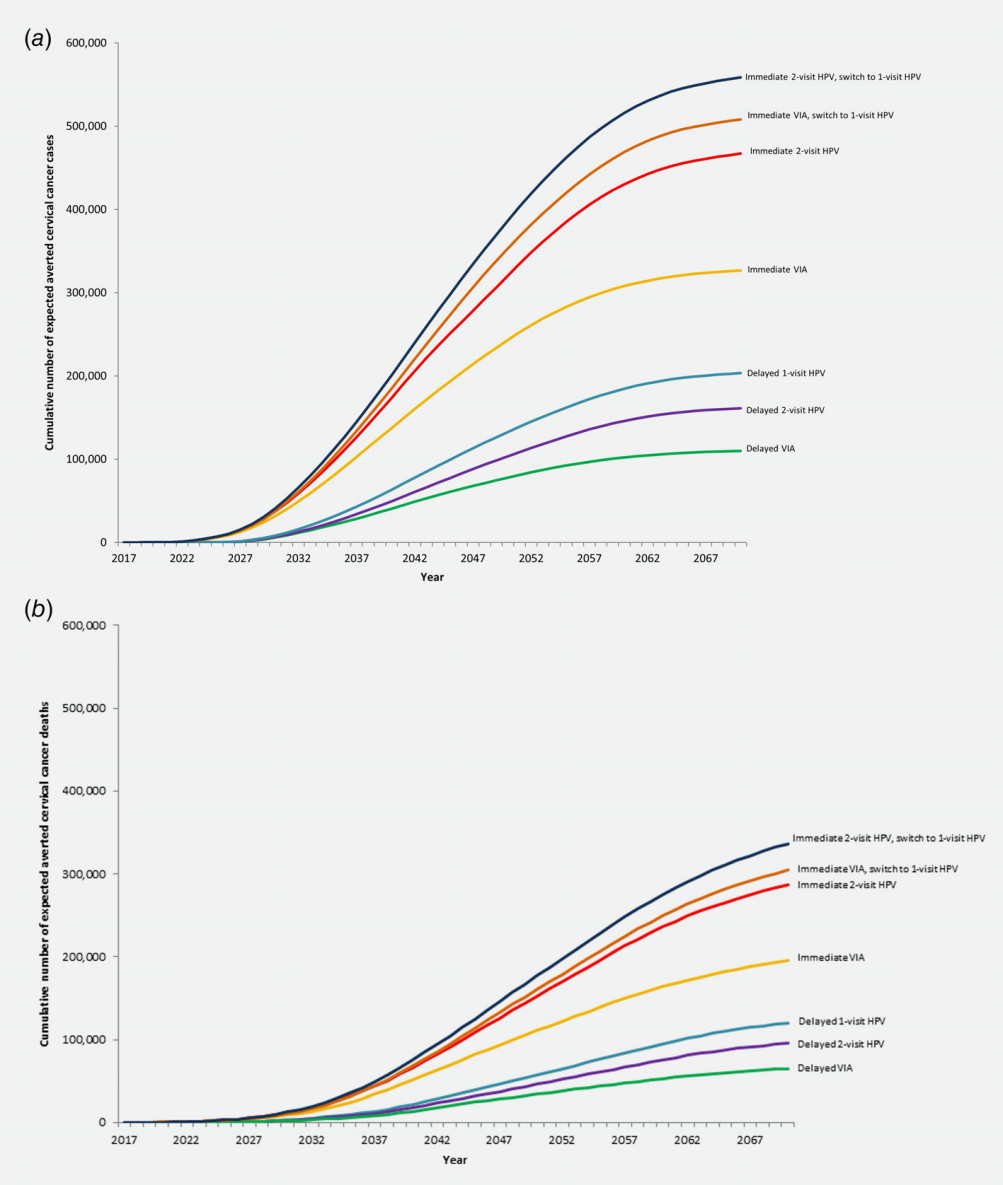
Cumulative number of expected averted cervical cancer (*a*) cases and (*b*) deaths are presented for the following screening strategies: (1) immediate two‐visit human papillomavirus (HPV) DNA testing followed by a switch to one‐visit HPV DNA testing after 5 years (dark blue line); (2) immediate visual inspection with acetic acid (VIA) followed by a switch to one‐visit HPV DNA testing after 5 years (orange line); (3) immediate two‐visit HPV DNA testing (red line); (4) immediate VIA (yellow line); (5) delayed one‐visit HPV DNA testing (turquoise line); (6) delayed two‐visit HPV DNA testing (purple line); and (7) delayed VIA (green line). Immediate implementation strategies for once in a lifetime screening assume 10% coverage each year of women aged 30–34 years in India between 2017 and 2021, and 20% coverage each year of women aged 30–34 years between 2022 and 2026. Delayed implementation strategies assume no coverage of women aged 30–34 years between 2017 and 2021, and 10% coverage each year of women aged 30–34 years between 2022 and 2026. The difference between the two‐visit and one‐visit HPV DNA testing strategies is that, in the two‐visit strategy, 20% of women screened do not show up to receive HPV results and, if HPV‐positive, treatment. In the one‐visit strategy, all women who screen positive and are eligible are assumed to receive treatment with cryotherapy.

Without screening, the CERVIVAC model projected 3,824,700 cervical cancer cases and 2,878,300 deaths over the lifetimes of women aged 30–34 years between 2017 and 2026 (equivalent to lifetime risks of 2.5% and 1.9%, respectively, of the 151 million women alive in these cohorts when each first became eligible for screening). The cumulative number of cervical cancer cases and deaths that could potentially be averted over the lifetimes of women in India aged 30–34 years during the intervention period (2017–2026) are presented in Table 3; the timing of cases and deaths averted, by calendar year, is displayed in Figure 1. Scenarios involving immediate implementation of screening in 2017 were predicted to avert substantially more cervical cancer cases and deaths than scenarios that delayed implementation until 2022. Immediate implementation of two‐visit HPV testing with a switch to one‐visit HPV testing was the most effective scenario, averting an estimated 574,100 cases and 382,500 deaths. Immediate implementation of VIA with a switch to one‐visit HPV testing was the next most effective scenario, and was projected to avert 521,900 cases and 346,600 deaths.

**Table 3:** Cervical cancer cases and deaths averted over the lifetimes of women aged 30–34 years during the intervention period 2017–2026 in India, by cervical cancer screening implementation strategy

Strategy^1^	Number of women screened	Cumulative cases averted	Cumulative deaths averted	Incremental number of cases averted relative to most effective strategy	Incremental number of deaths averted relative to most effective strategy
Immediate implementation (2017) followed by a switch to point‐of‐care HPV testing (2022)					
Immediate two‐visit HPV, switch to one‐visit HPV	81,377,000	574,100	382,500	–	–
Immediate VIA, switch to one‐visit HPV	81,377,000	521,900	346,600	−52,200	−35,900
Immediate implementation of currently available tests (2017) with moderate scale‐up (2022)					
Immediate two‐visit HPV	81,377,000	480,600	328,000	−93,500	−54,500
Immediate VIA	81,377,000	330,800	217,900	−243,300	−164,600
Delayed implementation of currently available tests or point‐of‐care HPV testing (2022)					
Delayed one‐visit HPV	27,435,000	209,300	139,100	−364,800	−243,400
Delayed two‐visit HPV	27,435,000	165,900	111,600	−408,200	−270,900
Delayed VIA	27,435,000	111,200	73,800	−462,900	−308,700

1 Strategies are listed in order of effectiveness. The target population included cohorts of women aged 30–34 years during implementation between 2017 and 2026. Cumulative cases and deaths extend over the lifetime of these cohorts, through 2095. Strategies considered for immediate implementation followed by a switch to point‐of‐care HPV testing included (1) Immediate two‐visit HPV, switch to one‐visit HPV: two‐visit HPV DNA testing with 10% coverage (implemented 2017–2021) scaling up to 20% coverage with one‐visit HPV testing (implemented 2022–2026); and (2) Immediate VIA, switch to one‐visit HPV: one‐visit VIA with 10% coverage (implemented 2017–2021) scaling up to 20% coverage with one‐visit HPV testing (implemented 2022–2026). Strategies considered for immediate implementation included (1) Immediate two‐visit HPV: two‐visit HPV DNA testing with 10% coverage (implemented 2017–2021) scaling up to 20% coverage (implemented 2022–2026); and (2) Immediate VIA: one‐visit VIA with 10% coverage (implemented 2017–2021) scaling up to 20% coverage (implemented 2022–2026). Strategies considered for delayed implementation included: (1) Delayed one‐visit HPV: one‐visit HPV DNA testing with 10% coverage (implemented 2022–2026); (2) Delayed two‐visit HPV: two‐visit HPV testing with 10% coverage (implemented 2022–2026); and (3) Delayed VIA: one‐visit VIA with 10% coverage (implemented 2022–2026).

Among immediate implementation strategies involving only currently available screening tests, immediate two‐visit HPV testing was more effective than immediate VIA, averting an estimated additional 149,800 cases and 110,100 deaths.

Despite comparable single‐visit delivery and more favorable test sensitivity to detect CIN2 and higher, delayed implementation of one‐visit HPV testing averted fewer cases and deaths than immediate implementation of VIA. Delayed implementation of one‐visit HPV testing averted 364,800 fewer cases and 243,400 fewer deaths than immediate two‐visit HPV with a switch to one‐visit HPV. Delayed implementation of two‐visit HPV testing and one‐visit VIA were projected to avert the fewest number of cases and deaths.

### Discussion

Our study supports the position that even short delays implementing organized screening programs in low‐resource settings with a high cervical cancer burden will result in substantial morbidity and loss of life. We used a model‐based approach—relying on both an individual‐based microsimulation model that captures the reduction in disease burden associated with nuanced screening algorithms and a population model—to project the number of cervical cancer cases and deaths that could be averted with immediate implementation of organized cervical cancer screening in India *versus* a five‐year delay in implementation. Our roll‐out assumptions were conservative, assuming only 10% of each birth cohort of women aged 30–34 years received screening in each of the first 5 years of implementation. For immediately implemented strategies, we assumed scale‐up to 20% coverage after 5 years (either continuing with currently available screening tests—VIA or two‐visit HPV—or introducing a new one‐visit HPV test). Among the strategies considered for delayed implementation was one‐visit HPV testing, to determine whether waiting for technological improvements (i.e., point‐of‐care HPV testing) can overcome the potential loss of life due to postponing roll‐out. Even under assumptions of low population coverage over the next 10 years, we projected that immediate implementation of two‐visit HPV testing with a switch to one‐visit HPV testing can avert 574,100 cases and 382,500 deaths in India relative to no screening. If implementation is delayed by 5 years to wait for an improved HPV test that facilitates same‐day screening and treatment of precancer, 364,800 fewer cases and 243,400 fewer deaths will be averted. Our model projections also indicate that even if a point‐of‐care test does not become widely available in 5 years, immediate implementation of two‐visit HPV testing with moderate scale‐up may still avert 480,600 cases and 328,000 deaths relative to no screening.

Three large randomized trials have demonstrated that screening can reduce cervical cancer incidence and mortality in India. In the Osmanabad district, a single round of HPV testing reduced advanced cervical cancer incidence and mortality by approximately 50%,^24^ while VIA did not. However, a single round of VIA in Dindigul district reduced incidence and mortality by 25% and 35%, respectively.^25^ Multiple rounds of VIA in Mumbai reduced cancer mortality by approximately 30%,^26^ but this was likely due to earlier detection of cancer rather than reduced incidence, and required extensive training of health workers. To successfully scale VIA, substantial training and quality control efforts will be needed to maintain test performance.

To date, HPV testing and VIA have generally been limited to clinical studies and demonstration projects in India.^2^ A notable exception is the Tamil Nadu Health Systems Project, which piloted a large‐scale government‐led VIA program in 2007. By 2015, the program achieved 71% coverage of women aged 30–60 years.^27^ While the program demonstrated the feasibility and acceptability of a cervical cancer screening program within the public health sector in one state, it also identified implementation challenges. Many of the women referred to treatment did not receive it, as women were referred to colposcopy at higher level facilities rather than receiving immediate treatment following a positive screening test.^28^ Furthermore, the project was supported by the World Bank (US$19 million for the noncommunicable disease program targeting cardiovascular disease, breast cancer and cervical cancer), highlighting the importance of multilateral institutions and the international donor community in strengthening health systems and building capacity to expand the reach of screening programs.

There are several limitations to our analysis. Our modeled screening algorithms assumed that currently available technologies could facilitate screening and treatment of precancer in one or two visits with VIA or HPV testing, respectively. While this may be the case at higher level facilities with cryotherapy available on‐site, the equipment and compressed gas needed for cryotherapy are not available at most primary health facilities in India. Thus, women may be required to bear the time and travel costs to attend a referral facility, reducing the likelihood of receiving treatment. However, the coverage rates we assumed are lower, covering a much narrower age range (30–34 years) than the 71% coverage achieved in the Tamil Nadu Health Systems Project for women aged 30–60 years, and might resemble program coverage rates if initial efforts focused on delivery of screen‐and‐treat algorithms in referral centers and/or mobile clinics to ensure on‐site availability of ablative treatment. While it is possible that delayed implementation could yield higher population coverage than immediate implementation, this would require very rapid deployment of resources and capacity building, as well as social mobilization and outreach. In India, where 67% of the population lives in rural areas,^29^ our assumptions of high compliance with treatment (100% with VIA; 80% with two‐visit HPV testing) among screen‐positive women eligible for cryotherapy are more likely to be achievable when the number of required visits for a screening episode is low and treatment is proximal. Furthermore, we did not consider the impact of catch‐up HPV vaccination in older women over age 30 years, for which the cost‐effectiveness has not been demonstrated in low‐resource settings.

The accuracy of microsimulation model projections depends upon the validity of our calibration approach. While we calibrated the model to fit epidemiologic data from India—using the expected value outcomes from 50 good‐fitting sets of natural history inputs to reflect uncertainty—the high‐risk HPV prevalence from a large demonstration project in Hyderabad^6^ and the Nagpur cancer registry^18^ do not fully capture the geographic variation in disease burden across India. Furthermore, our data on age‐specific prevalence of high‐risk HPV was restricted to women aged 30–49 years. However, exercises to validate model projections suggest internal consistency between our calibrated model, test performance characteristics for VIA and HPV testing drawn from a demonstration project in a public sector clinic in Hyderabad,^6^ and two large randomized trials of screening impact in India that were not used to derive model inputs (Appendix).^24^, ^25^ The calibrated and validated microsimulation model allowed us to estimate age‐specific cervical cancer incidence and mortality reductions resulting from nuanced screening and management algorithms, given uncertainty in the natural history parameters and epidemiologic data.

Our modeling approach assumed that the burden of cervical cancer was consistent over the lifetime of women aged 30–34 years between 2017 and 2026. A recent modeling analysis used age‐period‐cohort models to project the future number of new cervical cancer cases in six Baltic, central and eastern European countries through 2040.^30^ Based on recorded trends in the most recent generations, these projections assumed cervical cancer incidence continued to increase across the study period. It is unclear whether cervical cancer burden is also increasing in India, although HPV transmission models (which integrate sexual behavior data) might inform estimates of future cancer risk among young women at or approaching screening age. We note that if GLOBOCAN 2012 underestimates the future cancer risk of women who will become eligible for screening over the intervention period we consider, we may underestimate the number of cases averted due to organized screening.

The present analysis does not address the cost‐effectiveness or affordability of implementing a screening program, although both the value and budget impact need to be favorable in order for a program to be sustainable. Elsewhere, we have estimated the cost‐effectiveness of various screening strategies in India, and found that a two‐visit approach with HPV DNA testing three times in a woman's lifetime would be very cost‐effective, with an incremental cost‐effectiveness ratio below India's *per capita* GDP.^13^ We have found that the value of new technologies such as point‐of‐care HPV testing in India is potentially high if linkage to treatment can be assured.^31^ Furthermore, we have also estimated the financial cost of screening women aged 35 years in India with HPV testing or VIA once in a lifetime to be US$830 million over 10 years (or approximately US$83 million per year); however, this scenario represents an upper bound, as it assumed the program would reach full coverage within 5 years.^32^ To place this figure in context, US$1.08 billion of development assistance for health was disbursed to India in 2013.^33^ Decision makers will need to weigh the value and financial cost of implementing organized cervical cancer screening in relation to other disease priority areas. Development assistance will be required for screening implementation. We present these findings—which suggest that immediate implementation of HPV testing has the potential to save the lives of hundreds of thousands of women in India who are beyond the target age of HPV vaccination—to catalyze the policy dialog. This analysis quantifies the human cost of waiting to act, despite the availability of screening technologies with demonstrated effectiveness and cost‐effectiveness; this toll only increases over time.

Implementation of organized screening programs is difficult work, requiring political will and coordination across levels of the health care system. A one‐size‐fits‐all approach is unlikely to work across all states or districts within a state. Without injection of funds from the international donor community to strengthen health systems and support cancer prevention efforts, as well as governmental budgetary allocations and commitment to sustainability, the costs of cervical cancer screening will fall predominantly on women and their families; achieving high coverage and management of screen‐positive women will remain a barrier to successful programs. Despite these challenges, our study provides quantitative evidence that the failure to begin prompt implementation of cervical cancer screening in India with currently available technologies will result in substantial morbidity and mortality among women who are critical to social and economic stability.

### Ethics approval and consent to participate

Not applicable.

## Availability of data and materials

A Supporting Information provides details on microsimulation model inputs, calibration to epidemiologic data from India and calibration. The population model (CERVIVAC) has been described in this manuscript and elsewhere: Campos NG, Sharma M, Clark A, Lee L, Geng F, Regan C, Kim J, Resch R. The health and economic impact of scaling cervical cancer prevention in 50 low‐ and lower‐middle‐income countries. International Journal of Gynecology and Obstetrics 2017;138(S1):47–56. Available at: http://onlinelibrary.wiley.com/doi/10.1002/ijgo.12184/abstract.

## Author contributions

NGC contributed to study conceptualization, study design, data collection, data analysis, data interpretation, and drafted the manuscript. VT contributed to study conceptualization and revision of the manuscript. JJ contributed to study conceptualization and revision of the manuscript. CR contributed to data analysis, presentation of figures, and revision of the manuscript. SR contributed to study conceptualization, study design, data interpretation, and revision of the manuscript. AC contributed to programming of the CERVIVAC model, data collection, and revision of the manuscript. SS contributed to programming of the microsimulation model, data interpretation, and revision of the manuscript. JJK contributed to study conceptualization, study design, data interpretation, and revision of the manuscript.

## Supporting information


**Appendix S1**: The Population Health Impact of Delayed Implementation of Cervical Cancer Screening
