# Supplementary material for: Health impact of delayed implementation of cervical cancer screening programs in India: A modeling analysis
Source: Int J Cancer. 2018 Oct 16;144(4):687–96. doi: 10.1002/ijc.31823 (PMC6519250; doi:10.1002/ijc.31823)
Supplement: Supplementary file 1 — Appendix S1: The Population Health Impact of Delayed Implementation of Cervical Cancer Screening [file IJC-144-687-s001.docx]

**Appendix:**

**The Population Health Impact of Delayed Implementation of Cervical Cancer Screening Programs in India:**

**A modelling analysis**

This appendix provides additional details on methods, assumptions, and results presented in the main manuscript.

**MODEL CALIBRATION**

**Overview of the calibration process**

Details of the model development process, including initial parameterization and calibration, have been previously published.[^1^](#_ENREF_1)^,^ [^2^](#_ENREF_2) Derivation of model parameter values requires an iterative process involving comprehensive literature reviews, data synthesis and analysis, consultations with experts, and explorations of the influence of uncertain parameters and assumptions in the model. Baseline HPV incidence rates, as a function of genotype and age, were derived from published data from a prospective cohort of sexually active women aged 15-85 years in Bogota, Colombia.[^3^](#_ENREF_3) Because HPV incidence is known to vary by population as a function of sexual behaviors, age-specific HPV incidence and natural immunity following initial infection were considered important candidates for calibration. Transitions occurring from the HPV state (i.e., time-dependent rates of HPV clearance and progression by genotype) were informed by primary longitudinal data from the control arm of the Costa Rica Vaccine Trial.[^4^](#_ENREF_4) Type-specific data on CIN2 and CIN3 regression and progression are limited, [^5-10^](#_ENREF_5) so these parameters were also candidates for calibration. Because of the computational intensity of microsimulation models, we selected parameters for calibration based on the availability of 1) a range of plausible values and 2) good empirical data to inform calibration targets (i.e., high-risk HPV prevalence to calibrate HPV incidence rates; cancer incidence to calibrate CIN2 and CIN3 regression and progression rates).

To calibrate the model, we set plausible search ranges for multipliers to apply to baseline input values for age- and type-specific HPV incidence, as well as natural immunity following initial infection and progression and regression of CIN, and performed repeated model simulations in the absence of any preventive intervention. For each simulation, we randomly selected a single value for each of the multipliers from the identified plausible range, creating a unique vector of parameter values (i.e., parameter “set”). Following over 1,475,000 repeated samplings, we identified the parameter values with the highest correspondence to the empirical calibration target data by calculating and aggregating the log-likelihood of model-projected outcomes. We used the 50 parameter sets with the highest likelihood score (i.e., best overall fit to the empirical data) for analysis to capture uncertainty in the model parameters as a form of probabilistic sensitivity analysis. The mean reduction in lifetime risk of cervical cancer incidence across these top 50 parameter sets were used as inputs into the CERVIVAC population model.

**Calibration targets**

We assessed model fit by observing projected model outcomes of age-specific prevalence of high-risk HPV and age-specific cancer incidence relative to empirical data. The scoring algorithm included age-specific prevalence of high-risk HPV and age-specific cervical cancer incidence.

Age-specific prevalence of high-risk HPV was drawn from START-UP data on *care*HPV positivity using a cut-off ratio cut-point of 0.5 relative light units (**Table A.1**). For each age group, we derived a 95% binomial confidence interval around the point prevalence, which comprised the calibration target. The likelihood function for each age group was assumed to follow a binomial distribution.

Age-specific cancer incidence was drawn from the Nagpur registry in *Cancer in Five Continents*[^11^](#_ENREF_11) (**Table A.2**). The likelihood function for each age group was assumed to follow a normal distribution.

Composite goodness-of-fit scores for each input parameter set were generated by summing the log likelihood of each model outcome (i.e., age-specific HPV prevalence, age-specific cancer incidence). The 50 input parameter sets with the highest goodness-of-fit scores thus yielded the model outputs that were simultaneously closest to all calibration targets, and were selected for analysis. **Figures A.1-A.2** display model fit to epidemiologic data on age-specific prevalence of high-risk HPV and age-specific cancer incidence. Transition probabilities (including baseline input values and values of multipliers identified by the calibration process) are presented in **Table A.3.**

**Model validation exercises**

To assess the validity of the calibrated model, we compared the hazard ratios of advanced cervical cancer incidence and cervical cancer mortality predicted by onetime screening with VIA and HPV testing to the hazard ratios from a large randomized screening trial in India.[^12^](#_ENREF_12) In this exercise, we assumed screening took place at age 39 years (the median age of screening in the trial), and examined advanced cancer incidence and mortality at age 47 years (i.e., after 8 years of follow-up in the study) relative to no screening. We used test performance characteristics from the START-UP demonstration project in Hyderabad[^13^](#_ENREF_13) and management compliance data from the trial.[^12^](#_ENREF_12) Results are displayed in **Table A.4.** For HPV DNA testing, the hazard ratio predicted by the model was very close to the trial. For VIA, the trial found that advanced cancer incidence and mortality were not significantly different than in the control group, while our model found the hazard ratio of advanced cancer incidence relative to no screening was lower than the lower confidence bound suggested by the trial; the model hazard ratio for cancer mortality was within the confidence bounds suggested by the trial. However, the modelled hazard ratios for cancer incidence associated with VIA were more similar to a separate trial conducted in Tamil Nadu [^14^](#_ENREF_14).

**PROTOCOLS FOR TREATMENT OF PRECANCER**

Women who screened positive with either VIA or HPV DNA testing who were ineligible for cryotherapy were assumed to be referred to a secondary facility for colposcopy and subsequent treatment. Treatment protocols were based on information from in-country clinicians familiar with standard of care and availability of and preferences for treatment options. We assumed that, upon a histologic diagnosis of CIN1, CIN2, or CIN3, women received cryotherapy at a secondary facility. Following treatment of precancerous lesions with cryotherapy, we assumed women received follow-up at 1 year with cytology and colposcopy.

**REDUCTION IN CERVICAL CANCER INCIDENCE AND MORTALITY DUE TO SCREENING**

Age-specific percent reductions in the lifetime risk of cervical cancer incidence and mortality, relative to no screening, are presented in **Tables A.5-A.8** for each screening strategy and coverage level (i.e., 10%, 20%). These values were used as inputs into the CERVIVAC population model to project the cases and deaths averted due to implementation of each screening strategy over time.

**Table A.1. Age-specific prevalence of high-risk HPV, India**^a^**.**[**^13^**](#_ENREF_13)

| **Age group** | **Number of women** | **Number of women with high-risk HPV** | **Prevalence (95% CI)** |
| --- | --- | --- | --- |
| 30 – 34 years | 1,949 | 214 | 0.11 (0.10, 0.13) |
| 35 – 39 years | 1,158 | 99 | 0.09 (0.07, 0.10) |
| 40 – 44 years | 708 | 76 | 0.11 (0.09, 0.13) |
| 45 – 49 years | 687 | 85 | 0.12 (0.10, 0.15) |

^a^ HPV positivity was based on a cut-off of 0.5 relative light units.

**Table A.2. Age-specific cervical cancer incidence, India (Nagpur registry, 1998-2002)** ^a^**.**[**^11^**](#_ENREF_11)

| **Age group** | **Cases** | **Rate per 100,000 women (95% CI)** |
| --- | --- | --- |
| 20 – 24 years | 9 | 1.8 (0.6, 2.9) |
| 25 – 29 years | 11 | 2.3 (0.9, 3.6) |
| 30 – 34 years | 43 | 10.6 (7.4, 13.7) |
| 35 – 39 years | 62 | 16.9 (12.7, 21.1) |
| 40 – 44 years | 90 | 32.4 (25.7, 39.1) |
| 45 – 49 years | 107 | 46.2 (37.5, 55.0) |
| 50 – 54 years | 105 | 58.9 (47.6, 70.1) |
| 55 – 59 years | 70 | 52.4 (40.1, 64.7) |
| 60 – 64 years | 104 | 75.0 (60.6, 89.4) |
| 65 – 69 years | 71 | 62.5 (47.9, 77.0) |
| 70 – 74 years | 44 | 57.6 (40.6, 74.6) |
| ≥75 years | 9 | 26.8 (9.3, 44.2) |

^a^ Although our scoring algorithm included cancer incidence in women aged 30 to 49 years, we considered visual fit to all age groups.

**Table A.3. Monthly transition probabilities for the natural history model, India.**

| **Progression of Healthy to HPV** | | | | |
| --- | --- | --- | --- | --- |
| **HPV Type and Age Group, years** | Baseline Value | | Range of Multiplier Values Among Top 50 Parameter Sets | |
|  | Min | Max | Min | Max |
| **HPV 16** | | | | |
| <21 | 0 | 0.00186 | 0.301076 | 6.45143 |
| 21-24 | 0.000903 | 0.00125 | 0.300988 | 6.09928 |
| 25-29 | 0.000782 | 0.00087 | 0.300729 | 5.67089 |
| 30-49 | 0.000602 | 0.00078 | 0.316964 | 7.92735 |
| >= 50 | 0.000217 | 0.00059 | 0.312108 | 8.96237 |
|  |  |  |  |  |
|  |  |  |  |  |
| **HPV 18** | | | | |
| <21 | 0 | 0.00116 | 0.489594 | 9.79622 |
| 21-24 | 0.0011 | 0.001173 | 0.315438 | 9.61579 |
| 25-29 | 0.0006 | 0.001 | 0.34239 | 9.72271 |
| 30-49 | 0.000301 | 0.00058 | 0.333845 | 8.94467 |
| >= 50 | 0.000109 | 0.000295 | 0.310167 | 9.78527 |
|  |  |  |  |  |
| **HPV 31** | | | | |
| <21 | 0 | 0.001428 | 0.97413 | 7.98406 |
| 21-24 | 0.0009 | 0.001364 | 0.414976 | 7.7836 |
| 25-29 | 0.0006 | 0.000805 | 0.361407 | 6.24185 |
| 30-49 | 0.000301 | 0.00055 | 0.345243 | 7.66415 |
| >= 50 | 0.000109 | 0.000295 | 0.890981 | 7.88708 |
|  |  |  |  |  |
| **HPV 33** | | | | |
| <21 | 0.000786 | 0.000786 | 0.395009 | 5.7821 |
| 21-24 | 0.00069 | 0.00069 | 0.320448 | 5.66515 |
| 25-29 | 0.00036 | 0.00036 | 0.304039 | 5.45113 |
| 30-49 | 0.00022 | 0.00022 | 0.32892 | 5.85202 |
| >= 50 | 0.000128 | 0.000128 | 0.388383 | 5.98062 |
|  |  |  |  |  |
| **HPV 45** | | | | |
| <21 | 0 | 0.001 | 0.642692 | 7.93756 |
| 21-24 | 0.00041 | 0.0008 | 0.321723 | 6.16 |
| 25-29 | 0.000315 | 0.00037 | 0.334876 | 6.27828 |
| 30-49 | 0.000161 | 0.000305 | 0.305177 | 7.87527 |
| >= 50 | 0.000054 | 0.00015 | 0.346646 | 7.9058 |
|  |  |  |  |  |
| **HPV 52** | | | | |
| <21 | 0 | 0.001186 | 0.381688 | 5.92827 |
| 21-24 | 0.00027 | 0.0008 | 0.333476 | 4.18242 |
| 25-29 | 0.000184 | 0.000254 | 0.312324 | 4.76581 |
| 30-49 | 0.00015 | 0.000173 | 0.352 | 5.96265 |
| >= 50 | 0.000054 | 0.000148 | 0.683893 | 11.9166 |
|  |  |  |  |  |
| **HPV 58** | | | | |
| <21 | 0 | 0.001189 | 0.36424 | 7.96942 |
| 21-24 | 0.00059 | 0.0011 | 0.344115 | 6.92537 |
| 25-29 | 0.000465 | 0.00055 | 0.310383 | 7.57421 |
| 30-49 | 0.000244 | 0.00045 | 0.398455 | 7.99489 |
| >= 50 | 0.000054 | 0.000239 | 0.537506 | 7.67851 |
|  |  |  |  |  |
| **Other Oncogenic Types** | | | | |
| <21 | 0 | 0.00247 | 0.484726 | 5.98527 |
| 21-24 | 0.001804 | 0.0023 | 0.335258 | 5.61953 |
| 25-29 | 0.0012 | 0.0017 | 0.325255 | 5.48207 |
| 30-49 | 0.000602 | 0.0011 | 0.303116 | 5.65707 |
| >= 50 | 0.000217 | 0.00059 | 0.340359 | 5.8581 |
|  |  |  |  |  |
| **Nononcogenic Types** | | | | |
| <21 | 0 | 0.00261 | 0.370338 | 14.6661 |
| 21-24 | 0.00255 | 0.00262 | 0.311139 | 10.9989 |
| 25-29 | 0.002 | 0.0025 | 0.336878 | 12.6647 |
| 30-49 | 0.00085 | 0.00186 | 0.501555 | 14.3538 |
| >= 50 | 0.000217 | 0.0008 | 0.537661 | 14.6272 |

| **HPV Progression to CIN2** | | |
| --- | --- | --- |
| **HPV Type and Age Group, months** | Baseline Value | Multiplier Value Among Top 50 Parameter Sets |
| **HPV 16** |  |  |
| 1-15 | 0.001707 | 1 |
| 16-27 | 0.002422 | 1 |
| 28-39 | 0.002577 | 1 |
| 40-51 | 0.005518 | 1 |
| 52+ | 0.014998 | 1 |
|  |  |  |
| **HPV 18** |  |  |
| 1-15 | 0.0000427 | 1 |
| 16-27 | 0.000189 | 1 |
| 28-39 | 0.000189 | 1 |
| 40-51 | 0.007733 | 1 |
| 52+ | 0.007733 | 1 |
|  |  |  |
| **HPV 31** |  |  |
| 1-15 | 0.00026194 | 1 |
| 16-27 | 0.00278007 | 1 |
| 28-39 | 0.00309101 | 1 |
| 40-51 | 0.00693095 | 1 |
| 52+ | 0.00693095 | 1 |
|  |  |  |
| **HPV 33** |  |  |
| 1-15 | 0.00071924 | 1 |
| 16-27 | 0.00071924 | 1 |
| 28-39 | 0.00493869 | 1 |
| 40-51 | 0.00493869 | 1 |
| 52+ | 0.00493869 | 1 |
|  |  |  |
| **HPV 45** |  |  |
| 1-15 | 0 | 1 |
| 16-27 | 0 | 1 |
| 28-39 | 0.00225755 | 1 |
| 40-51 | 0.00533318 | 1 |
| 52+ | 0.00533318 | 1 |
|  |  |  |
| **HPV 52** |  |  |
| 1-15 | 0.00088413 | 1 |
| 16-27 | 0.00168129 | 1 |
| 28-39 | 0.00168129 | 1 |
| 40-51 | 0.00197781 | 1 |
| 52+ | 0.00567833 | 1 |
|  |  |  |
| **HPV 58** |  |  |
| 1-15 | 0.00058707 | 1 |
| 16-27 | 0.00246817 | 1 |
| 28-39 | 0.00246817 | 1 |
| 40-51 | 0.00461685 | 1 |
| 52+ | 0.01024565 | 1 |
|  |  |  |
| **High Risk HPV** |  |  |
| 1-15 | 0.00012644 | 1 |
| 16-27 | 0.00037279 | 1 |
| 28-39 | 0.0019601 | 1 |
| 40-51 | 0.0019601 | 1 |
| 52+ | 0.0019601 | 1 |
|  |  |  |
| **Low Risk HPV** |  |  |
| 1-15 | 0.0002052 | 1 |
| 16-27 | 0.00029071 | 1 |
| 28-39 | 0.00030961 | 1 |
| 40-51 | 0.00066332 | 1 |
| 52+ | 0.00066332 | 1 |
|  |  |  |
| **HPV progression to CIN3** | | |
| **HPV Type and Age Group, months** | Baseline Value | Multiplier Value Among Top 50 Parameter Sets |
| **HPV 16** |  |  |
| 1-15 | 0.000569 | 1 |
| 16-27 | 0.000808 | 1 |
| 28-39 | 0.00086 | 1 |
| 40-51 | 0.001843 | 1 |
| 52+ | 0.005024 | 1 |
|  |  |  |
| **HPV 18** |  |  |
| 1-15 | 0.0000107 | 1 |
| 16-27 | 0.0000472 | 1 |
| 28-39 | 0.0000472 | 1 |
| 40-51 | 0.001939 | 1 |
| 52+ | 0.001939 | 1 |
|  |  |  |
| **HPV 31** |  |  |
| 1-15 | 6.5492E-05 | 1 |
| 16-27 | 0.00069574 | 1 |
| 28-39 | 0.00077365 | 1 |
| 40-51 | 0.00173726 | 1 |
| 52+ | 0.00173726 | 1 |
|  |  |  |
| **HPV 33** |  |  |
| 1-15 | 0.00017986 | 1 |
| 16-27 | 0.00017986 | 1 |
| 28-39 | 0.00123696 | 1 |
| 40-51 | 0.00123696 | 1 |
| 52+ | 0.00123696 | 1 |
|  |  |  |
| **HPV 45** |  |  |
| 1-15 | 0 | 1 |
| 16-27 | 0 | 1 |
| 28-39 | 0.00056487 | 1 |
| 40-51 | 0.00133597 | 1 |
| 52+ | 0.00133597 | 1 |
|  |  |  |
| **HPV 52** |  |  |
| 1-15 | 0.00022111 | 1 |
| 16-27 | 0.00042059 | 1 |
| 28-39 | 0.00042059 | 1 |
| 40-51 | 0.00049482 | 1 |
| 52+ | 0.00142262 | 1 |
|  |  |  |
| **HPV 58** |  |  |
| 1-15 | 0.0001468 | 1 |
| 16-27 | 0.00061761 | 1 |
| 28-39 | 0.00061761 | 1 |
| 40-51 | 0.00115622 | 1 |
| 52+ | 0.00257131 | 1 |
|  |  |  |
| **High Risk HPV** |  |  |
| 1-15 | 3.1611E-05 | 1 |
| 16-27 | 9.3211E-05 | 1 |
| 28-39 | 0.00049039 | 1 |
| 40-51 | 0.00049039 | 1 |
| 52+ | 0.00049039 | 1 |
|  |  |  |
| **Low Risk HPV** |  |  |
| 1-15 | 2.2802E-05 | 1 |
| 16-27 | 3.2305E-05 | 1 |
| 28-39 | 3.4405E-05 | 1 |
| 40-51 | 7.3724E-05 | 1 |
| 52+ | 7.3724E-05 | 1 |

| **Progression of CIN2 to CA** | | | | |  |  |
| --- | --- | --- | --- | --- | --- | --- |
| **HPV Type and Age Group, years** | Baseline Value | Range of Multiplier Values Among Top 50 Parameter Sets | | |  |  |
|  |  | Min | | Max |  |  |
| **HPV16** |  |  | |  |  |  |
| 1-5 | 0.00003294 | 1.00001 | | 1.49731 |  |  |
| 6-10 | 0.00003564 | 1.00001 | | 1.49731 |  |  |
| 11-20 | 0.0008568 | 1.00001 | | 1.49731 |  |  |
| 21-29 | 0.0025056 | 1.00001 | | 1.49731 |  |  |
| 30-34 | 0.0050112 | 1.00001 | | 1.49731 |  |  |
| 35-39 | 0.0054288 | 1.00001 | | 1.49731 |  |  |
| 40-44 | 0.0116928 | 1.00001 | | 1.49731 |  |  |
| 45-49 | 0.012528 | 1.00001 | | 1.49731 |  |  |
| 50+ | 0.33408 | 1.00001 | | 1.49731 |  |  |
|  |  |  | |  |  |  |
| **HPV 18** |  |  | |  |  |  |
| 1-5 | 0.00003294 | 1.00001 | | 1.49731 |  |  |
| 6-10 | 0.00003564 | 1.00001 | | 1.49731 |  |  |
| 11-20 | 0.0005712 | 1.00001 | | 1.49731 |  |  |
| 21-29 | 0.0016704 | 1.00001 | | 1.49731 |  |  |
| 30-34 | 0.0033408 | 1.00001 | | 1.49731 |  |  |
| 35-39 | 0.0036192 | 1.00001 | | 1.49731 |  |  |
| 40-44 | 0.0077952 | 1.00001 | | 1.49731 |  |  |
| 45-49 | 0.008352 | 1.00001 | | 1.49731 |  |  |
| 50+ | 0.22272 | 1.00001 | | 1.49731 |  |  |
|  |  |  | |  |  |  |
| **HPV 31** |  |  | |  |  |  |
| 1-5 | 0.00002196 | 1.00001 | | 1.49731 |  |  |
| 6-10 | 0.0000238 | 1.00001 | | 1.49731 |  |  |
| 11-20 | 0.0008568 | 1.00001 | | 1.49731 |  |  |
| 21-29 | 0.0025056 | 1.00001 | | 1.49731 |  |  |
| 30-34 | 0.0050112 | 1.00001 | | 1.49731 |  |  |
| 35-39 | 0.0054288 | 1.00001 | | 1.49731 |  |  |
| 40-44 | 0.0116928 | 1.00001 | | 1.49731 |  |  |
| 45-49 | 0.012528 | 1.00001 | | 1.49731 |  |  |
| 50+ | 0.33408 | 1.00001 | | 1.49731 |  |  |
|  |  |  | |  |  |  |
| **HPV 33** |  |  | |  |  |  |
| 1-5 | 0.00003294 | 1.00001 | | 1.49731 |  |  |
| 6-10 | 0.0000356 | 1.00001 | | 1.49731 |  |  |
| 11-20 | 0.0005712 | 1.00001 | | 1.49731 |  |  |
| 21-29 | 0.0016704 | 1.00001 | | 1.49731 |  |  |
| 30-34 | 0.0033408 | 1.00001 | | 1.49731 |  |  |
| 35-39 | 0.0036192 | 1.00001 | | 1.49731 |  |  |
| 40-44 | 0.0077952 | 1.00001 | | 1.49731 |  |  |
| 45-49 | 0.008352 | 1.00001 | | 1.49731 |  |  |
| 50+ | 0.22272 | 1.00001 | | 1.49731 |  |  |
|  |  |  | |  |  |  |
| **HPV 45** |  |  | |  |  |  |
| 1-5 | 0.00002196 | 1.00001 | | 1.49731 |  |  |
| 6-10 | 0.0000238 | 1.00001 | | 1.49731 |  |  |
| 11-20 | 0.0005712 | 1.00001 | | 1.49731 |  |  |
| 21-29 | 0.0016704 | 1.00001 | | 1.49731 |  |  |
| 30-34 | 0.0033408 | 1.00001 | | 1.49731 |  |  |
| 35-39 | 0.0036192 | 1.00001 | | 1.49731 |  |  |
| 40-44 | 0.0077952 | 1.00001 | | 1.49731 |  |  |
| 45-49 | 0.008352 | 1.00001 | | 1.49731 |  |  |
| 50+ | 0.22272 | 1.00001 | | 1.49731 |  |  |
|  |  |  | |  |  |  |
| **HPV 52** |  |  | |  |  |  |
| 1-5 | 0.00002196 | 1.00001 | | 1.49731 |  |  |
| 6-10 | 0.0000238 | 1.00001 | | 1.49731 |  |  |
| 11-20 | 0.0005712 | 1.00001 | | 1.49731 |  |  |
| 21-29 | 0.0016704 | 1.00001 | | 1.49731 |  |  |
| 30-34 | 0.0033408 | 1.00001 | | 1.49731 |  |  |
| 35-39 | 0.0036192 | 1.00001 | | 1.49731 |  |  |
| 40-44 | 0.0077952 | 1.00001 | | 1.49731 |  |  |
| 45-49 | 0.008352 | 1.00001 | | 1.49731 |  |  |
| 50+ | 0.22272 | 1.00001 | | 1.49731 |  |  |
|  |  |  | |  |  |  |
| **HPV 58** |  |  | |  |  |  |
| 1-5 | 0.00002196 | 1.00001 | | 1.49731 |  |  |
| 6-10 | 2.38E-05 | 1.00001 | | 1.49731 |  |  |
| 11-20 | 0.0005712 | 1.00001 | | 1.49731 |  |  |
| 21-29 | 0.0016704 | 1.00001 | | 1.49731 |  |  |
| 30-34 | 0.0033408 | 1.00001 | | 1.49731 |  |  |
| 35-39 | 0.0036192 | 1.00001 | | 1.49731 |  |  |
| 40-44 | 0.0077952 | 1.00001 | | 1.49731 |  |  |
| 45-49 | 0.008352 | 1.00001 | | 1.49731 |  |  |
| 50+ | 0.22272 | 1.00001 | | 1.49731 |  |  |
|  |  |  | |  |  |  |
| **High Risk HPV** |  |  | |  |  |  |
| 1-5 | 0.00002196 | 1.00001 | | 1.49731 |  |  |
| 6-10 | 0.00002376 | 1.00001 | | 1.49731 |  |  |
| 11-20 | 0.0008568 | 1.00001 | | 1.49731 |  |  |
| 21-29 | 0.0025056 | 1.00001 | | 1.49731 |  |  |
| 30-34 | 0.0050112 | 1.00001 | | 1.49731 |  |  |
| 35-39 | 0.0054288 | 1.00001 | | 1.49731 |  |  |
| 40-44 | 0.0116928 | 1.00001 | | 1.49731 |  |  |
| 45-49 | 0.012528 | 1.00001 | | 1.49731 |  |  |
| 50+ | 0.33408 | 1.00001 | | 1.49731 |  |  |
|  |  |  | |  |  |  |
| **Progression of CIN3 to CA** | | | | |  |  |
| **HPV Type and Age Group, years** | Baseline Value | Range of Multiplier Values Among Top 50 Parameter Sets | | |  |  |
|  |  | Min | | Max |  |  |
| **HPV 16** |  |  | |  |  |  |
| 1-5 | 0.0001647 | 1.00426 | | 1.46295 |  |  |
| 6-10 | 0.0001782 | 1.00426 | | 1.46295 |  |  |
| 11-20 | 0.004284 | 1.00426 | | 1.46295 |  |  |
| 21-29 | 0.012528 | 1.00426 | | 1.46295 |  |  |
| 30-34 | 0.025056 | 1.00426 | | 1.46295 |  |  |
| 35-39 | 0.027144 | 1.00426 | | 1.46295 |  |  |
| 40-44 | 0.058464 | 1.00426 | | 1.46295 |  |  |
| 45-49 | 0.06264 | 1.00426 | | 1.46295 |  |  |
| 50+ | 0.33408 | 1.00426 | | 1.46295 |  |  |
|  |  |  | |  |  |  |
| **HPV 18** |  |  | |  |  |  |
| 1-5 | 0.0001647 | 1.00426 | | 1.46295 |  |  |
| 6-10 | 0.0001782 | 1.00426 | | 1.46295 |  |  |
| 11-20 | 0.004284 | 1.00426 | | 1.46295 |  |  |
| 21-29 | 0.012528 | 1.00426 | | 1.46295 |  |  |
| 30-34 | 0.025056 | 1.00426 | | 1.46295 |  |  |
| 35-39 | 0.027144 | 1.00426 | | 1.46295 |  |  |
| 40-44 | 0.058464 | 1.00426 | | 1.46295 |  |  |
| 45-49 | 0.06264 | 1.00426 | | 1.46295 |  |  |
| 50+ | 0.33408 | 1.00426 | | 1.46295 |  |  |
|  |  |  | |  |  |  |
| **HPV 31** |  |  | |  |  |  |
| 1-5 | 0.0001098 | 1.00426 | | 1.46295 |  |  |
| 6-10 | 0.0001188 | 1.00426 | | 1.46295 |  |  |
| 11-20 | 0.002856 | 1.00426 | | 1.46295 |  |  |
| 21-29 | 0.008352 | 1.00426 | | 1.46295 |  |  |
| 30-34 | 0.016704 | 1.00426 | | 1.46295 |  |  |
| 35-39 | 0.018096 | 1.00426 | | 1.46295 |  |  |
| 40-44 | 0.038976 | 1.00426 | | 1.46295 |  |  |
| 45-49 | 0.04176 | 1.00426 | | 1.46295 |  |  |
| 50+ | 0.22272 | 1.00426 | | 1.46295 |  |  |
|  |  |  | |  |  |  |
| **HPV 33** |  |  | |  |  |  |
| 1-5 | 0.0001647 | 1.00426 | | 1.46295 |  |  |
| 6-10 | 0.0001782 | 1.00426 | | 1.46295 |  |  |
| 11-20 | 0.004284 | 1.00426 | | 1.46295 |  |  |
| 21-29 | 0.012528 | 1.00426 | | 1.46295 |  |  |
| 30-34 | 0.025056 | 1.00426 | | 1.46295 |  |  |
| 35-39 | 0.027144 | 1.00426 | | 1.46295 |  |  |
| 40-44 | 0.058464 | 1.00426 | | 1.46295 |  |  |
| 45-49 | 0.06264 | 1.00426 | | 1.46295 |  |  |
| 50+ | 0.33408 | 1.00426 | | 1.46295 |  |  |
|  |  |  | |  |  |  |
| **HPV 45** |  |  | |  |  |  |
| 1-5 | 0.0001098 | 1.00426 | | 1.46295 |  |  |
| 6-10 | 0.0001188 | 1.00426 | | 1.46295 |  |  |
| 11-20 | 0.002856 | 1.00426 | | 1.46295 |  |  |
| 21-29 | 0.008352 | 1.00426 | | 1.46295 |  |  |
| 30-34 | 0.016704 | 1.00426 | | 1.46295 |  |  |
| 35-39 | 0.018096 | 1.00426 | | 1.46295 |  |  |
| 40-44 | 0.038976 | 1.00426 | | 1.46295 |  |  |
| 45-49 | 0.04176 | 1.00426 | | 1.46295 |  |  |
| 50+ | 0.22272 | 1.00426 | | 1.46295 |  |  |
|  |  |  | |  |  |  |
| **HPV 52** |  |  | |  |  |  |
| 1-5 | 0.0001098 | 1.00426 | | 1.46295 |  |  |
| 6-10 | 0.0001188 | 1.00426 | | 1.46295 |  |  |
| 11-20 | 0.002856 | 1.00426 | | 1.46295 |  |  |
| 21-29 | 0.008352 | 1.00426 | | 1.46295 |  |  |
| 30-34 | 0.016704 | 1.00426 | | 1.46295 |  |  |
| 35-39 | 0.018096 | 1.00426 | | 1.46295 |  |  |
| 40-44 | 0.038976 | 1.00426 | | 1.46295 |  |  |
| 45-49 | 0.04176 | 1.00426 | | 1.46295 |  |  |
| 50+ | 0.22272 | 1.00426 | | 1.46295 |  |  |
|  |  |  | |  |  |  |
| **HPV 58** |  |  | |  |  |  |
| 1-5 | 0.0001098 | 1.00426 | | 1.46295 |  |  |
| 6-10 | 0.0001188 | 1.00426 | | 1.46295 |  |  |
| 11-20 | 0.002856 | 1.00426 | | 1.46295 |  |  |
| 21-29 | 0.008352 | 1.00426 | | 1.46295 |  |  |
| 30-34 | 0.016704 | 1.00426 | | 1.46295 |  |  |
| 35-39 | 0.018096 | 1.00426 | | 1.46295 |  |  |
| 40-44 | 0.038976 | 1.00426 | | 1.46295 |  |  |
| 45-49 | 0.04176 | 1.00426 | | 1.46295 |  |  |
| 50+ | 0.22272 | 1.00426 | | 1.46295 |  |  |
|  |  |  | |  |  |  |
| **High Risk HPV** |  |  | |  |  |  |
| 1-5 | 0.0001098 | 1.00426 | | 1.46295 |  |  |
| 6-10 | 0.0001188 | 1.00426 | | 1.46295 |  |  |
| 11-20 | 0.002856 | 1.00426 | | 1.46295 |  |  |
| 21-29 | 0.008352 | 1.00426 | | 1.46295 |  |  |
| 30-34 | 0.016704 | 1.00426 | | 1.46295 |  |  |
| 35-39 | 0.018096 | 1.00426 | | 1.46295 |  |  |
| 40-44 | 0.038976 | 1.00426 | | 1.46295 |  |  |
| 45-49 | 0.04176 | 1.00426 | | 1.46295 |  |  |
| 50+ | 0.22272 | 1.00426 | | 1.46295 |  |  |
| **Progression of invasive cancer stages** | | | | | |  |
|  | | | **Baseline Value** | | |  |
| Local to regional | | | 0.02 | | |  |
| Regional to distant | | | 0.025 | | |  |
|  | | |  | | |  |
| **Invasive cancer mortality** | | |  | | |  |
| Local | | | 0.005876 | | |  |
| Regional | | | 0.0151371 | | |  |
| Distant | | | 0.0655189 | | |  |
|  | | |  | | |  |
| **Probability of symptom detection** | | |  | | |  |
| Local | | | 0.0039 | | |  |
| Regional | | | 0.1333 | | |  |
| Distant | | | 0.1746 | | |  |
|  | | |  | | |  |

| **HPV clearance** | | |
| --- | --- | --- |
| **HPV Type and Age Group, months** | Baseline Value | Multiplier Value Among Top 50 Parameter Sets |
| **HPV 16** |  |  |
| 1-15 | 0.041886 | 1 |
| 16-27 | 0.040754 | 1 |
| 28-39 | 0.033905 | 1 |
| 40-51 | 0.031888 | 1 |
| 52+ | 0.019846 | 1 |
|  |  |  |
| **HPV 18** |  |  |
| 1-15 | 0.073342 | 1 |
| 16-27 | 0.063235 | 1 |
| 28-39 | 0.053605 | 1 |
| 40-51 | 0.020616 | 1 |
| 52+ | 0.020616 | 1 |
|  |  |  |
| **HPV 31** |  |  |
| 1-15 | 0.063447 | 1 |
| 16-27 | 0.033826 | 1 |
| 28-39 | 0.033826 | 1 |
| 40-51 | 0.033826 | 1 |
| 52+ | 0.033826 | 1 |
|  |  |  |
| **HPV 33** |  |  |
| 1-15 | 0.083452 | 1 |
| 16-27 | 0.044955 | 1 |
| 28-39 | 0.036156 | 1 |
| 40-51 | 0.036156 | 1 |
| 52+ | 0.036156 | 1 |
|  |  |  |
| **HPV 45** |  |  |
| 1-15 | 0.078517 | 1 |
| 16-27 | 0.042579 | 1 |
| 28-39 | 0.041675 | 1 |
| 40-51 | 0.030133 | 1 |
| 52+ | 0.030133 | 1 |
|  |  |  |
| **HPV 52** |  |  |
| 1-15 | 0.062999 | 1 |
| 16-27 | 0.044401 | 1 |
| 28-39 | 0.044401 | 1 |
| 40-51 | 0.039325 | 1 |
| 52+ | 0.039325 | 1 |
|  |  |  |
| **HPV 58** |  |  |
| 1-15 | 0.065572 | 1 |
| 16-27 | 0.05443 | 1 |
| 28-39 | 0.053968 | 1 |
| 40-51 | 0.033319 | 1 |
| 52+ | 0.033319 | 1 |
|  |  |  |
| **High Risk HPV** |  |  |
| 1-15 | 0.080766 | 1 |
| 16-27 | 0.066633 | 1 |
| 28-39 | 0.053972 | 1 |
| 40-51 | 0.049229 | 1 |
| 52+ | 0.005094 | 1 |
|  |  |  |
| **Low Risk HPV** |  |  |
| 1-15 | 0.051888 | 1 |
| 16-27 | 0.050005 | 1 |
| 28-39 | 0.034649 | 1 |
| 40-51 | 0.034649 | 1 |
| 52-63 | 0.034649 | 1 |
| 64+ | 0.028608 | 1 |

| **Regression of CIN2 to Healthy** | | | | |
| --- | --- | --- | --- | --- |
| **HPV Type and Age Group, months** | Baseline Value | | Range of Multiplier Values Among Top 50 Parameter Sets | |
|  | Min | Max | Min | Max |
| **HPV 16** |  |  |  |  |
| 1-5 | 0.05 | 0.523578 | 0.500049 | 3.12354 |
| 6-10 | 0.035 | 0.523578 | 0.500049 | 3.12354 |
| 11-20 | 0.013 | 0.523578 | 0.500049 | 3.12354 |
| 21-29 | 0.0005 | 0.523578 | 0.500049 | 3.12354 |
| 30-39 | 0.0001 | 0.523578 | 0.500049 | 3.12354 |
| 40+ | 0.00005 | 0.523578 | 0.500049 | 3.12354 |
|  |  |  |  |  |
| **All Other HPV Types** |  |  |  |  |
| 1-5 | 0.05 | 0.523578 | 0.506455 | 3.84646 |
| 6-10 | 0.035 | 0.523578 | 0.506455 | 3.84646 |
| 11-20 | 0.013 | 0.523578 | 0.506455 | 3.84646 |
| 21-29 | 0.0005 | 0.523578 | 0.506455 | 3.84646 |
| 30-39 | 0.0001 | 0.523578 | 0.506455 | 3.84646 |
| 40+ | 0.00005 | 0.523578 | 0.506455 | 3.84646 |
|  | | | | |
| **Regression of CIN3 to Healthy** | | | | |
| **HPV Type and Age Group, months** | Baseline Value | | Range of Multiplier Values Among Top 50 Parameter Sets | |
|  | Min | Max | Min | Max |
| **HPV 16** |  |  |  |  |
| 1-5 | 0.025 | 0.639221 | 0.503246 | 3.80807 |
| 6-10 | 0.0175 | 0.639221 | 0.503246 | 3.80807 |
| 11-20 | 0.0065 | 0.639221 | 0.503246 | 3.80807 |
| 21-29 | 0.00025 | 0.639221 | 0.503246 | 3.80807 |
| 30-39 | 0.00005 | 0.639221 | 0.503246 | 3.80807 |
| 40+ | 0.000025 | 0.639221 | 0.503246 | 3.80807 |
|  |  |  |  |  |
| **All Other HPV Types** |  |  |  |  |
| 1-5 | 0.025 | 0.639221 | 0.53548 | 3.99878 |
| 6-10 | 0.0175 | 0.639221 | 0.53548 | 3.99878 |
| 11-20 | 0.0065 | 0.639221 | 0.53548 | 3.99878 |
| 21-29 | 0.00025 | 0.639221 | 0.53548 | 3.99878 |
| 30-39 | 0.00005 | 0.639221 | 0.53548 | 3.99878 |
| 40+ | 0.000025 | 0.639221 | 0.53548 | 3.99878 |

**Table A.4. Model validation results.**

| **Hazard ratio** | **Trial, HPV DNA testing** [**^12^**](#_ENREF_12)  **(95% CI)** | **Model, HPV DNA testing** | **Trial, VIA** [**^12^**](#_ENREF_12) **(95% CI)** | **Model, VIA** |
| --- | --- | --- | --- | --- |
| Advanced cervical cancer incidence | 0.47  (0.32, 0.69) | 0.45 | 1.04  (0.72, 1.49) | 0.67 |
| Cervical cancer mortality | 0.52  (0.33, 0.83) | 0.58 | 0.86  (0.6, 1.25) | 0.74 |

**Table A.5. Age-specific cervical cancer incidence reduction (%) relative to no screening, by age at screening and screening strategy, at 10% coverage.**^a^

| Age  (years) | Screening at age 30 years | | | Screening at age 31 years | | | Screening at age 32 years | | | Screening at age 33 years | | | Screening at age 34 years | | |
| --- | --- | --- | --- | --- | --- | --- | --- | --- | --- | --- | --- | --- | --- | --- | --- |
|  | VIA 1-v | HPV 2-v | HPV 1-v | VIA 1-v | HPV 2-v | HPV 1-v | VIA 1-v | HPV 2-v | HPV 1-v | VIA 1-v | HPV 2-v | HPV 1-v | VIA 1-v | HPV 2-v | HPV 1-v |
| 20-24 | 0.0 | 0.0 | 0.0 | 0.0 | 0.0 | 0.0 | 0.0 | 0.0 | 0.0 | 0.0 | 0.0 | 0.0 | 0.0 | 0.0 | 0.0 |
| 25-29 | 0.0 | 0.0 | 0.0 | 0.0 | 0.0 | 0.0 | 0.0 | 0.0 | 0.0 | 0.0 | 0.0 | 0.0 | 0.0 | 0.0 | 0.0 |
| 30-34 | 0.8 | 2.0 | 2.4 | 0.0^b^ | 0.0 | 0.4 | 0.0^b^ | 0.0^b^ | 0.0^b^ | 0.0^b^ | 0.0^b^ | 0.0^b^ | 0.0^b^ | 0.0^b^ | 0.0^b^ |
| 35-39 | 3.8 | 4.7 | 5.7 | 4.0 | 4.3 | 5.3 | 4.3 | 4.2 | 5.3 | 4.2 | 4.2 | 5.1 | 4.2 | 4.2 | 5.2 |
| 40-44 | 2.9 | 3.6 | 4.7 | 2.9 | 3.7 | 4.8 | 3.2 | 3.9 | 5.2 | 3.3 | 4.5 | 5.7 | 3.4 | 4.6 | 5.7 |
| 45-49 | 2.4 | 3.0 | 4.1 | 2.4 | 3.3 | 4.3 | 2.6 | 3.4 | 4.4 | 2.9 | 3.8 | 5.0 | 3.1 | 3.8 | 5.0 |
| 50-54 | 1.7 | 2.6 | 3.3 | 1.7 | 2.8 | 3.5 | 1.8 | 2.8 | 3.6 | 1.9 | 2.9 | 3.7 | 2.1 | 3.1 | 3.9 |
| 55-59 | 1.5 | 2.2 | 2.9 | 1.5 | 2.5 | 3.0 | 1.6 | 2.3 | 3.0 | 1.7 | 2.5 | 3.1 | 1.8 | 2.7 | 3.4 |
| 60-64 | 1.2 | 2.2 | 2.6 | 1.2 | 2.4 | 2.8 | 1.3 | 2.4 | 2.9 | 1.3 | 2.6 | 3.1 | 1.4 | 2.6 | 3.2 |
| 65-69 | 0.8 | 1.5 | 1.9 | 0.9 | 1.7 | 2.1 | 0.9 | 2.0 | 2.4 | 1.0 | 1.9 | 2.5 | 0.9 | 2.1 | 2.6 |
| 70-74 | 0.6 | 1.1 | 1.3 | 0.7 | 1.2 | 1.4 | 0.7 | 1.2 | 1.4 | 0.8 | 1.3 | 1.6 | 0.9 | 1.4 | 1.7 |
| 75-79 | 0.2 | 0.6 | 0.7 | 0.2 | 0.7 | 0.9 | 0.2 | 0.7 | 0.9 | 0.2 | 0.7 | 0.9 | 0.2 | 0.9 | 1.2 |

| ^a^ HPV 1-v: 1-visit HPV DNA testing; HPV 2-v: 2-visit HPV DNA testing; VIA 1-v: 1-visit visual inspection with acetic acid. Reductions at age 75 to 79 years were also applied to women aged 80 to 100 years in CERVIVAC.  ^b^ Where noted, cancer reductions estimated by the model were slightly negative, because an increase in the rate of detected cancers associated with screening outweighed the benefits of screening. In these instances, we assumed cancer reductions were zero to avoid biasing against screening. |
| --- |
|  |
|  |

**Table A.6. Age-specific cervical cancer incidence reduction (%) relative to no screening, by age at screening and screening strategy, at 20% coverage.**^a^

| Age  (years) | Screening at age 30 years | | | Screening at age 31 years | | | Screening at age 32 years | | | Screening at age 33 years | | | Screening at age 34 years | | |
| --- | --- | --- | --- | --- | --- | --- | --- | --- | --- | --- | --- | --- | --- | --- | --- |
|  | VIA 1-v | HPV 2-v | HPV 1-v | VIA 1-v | HPV 2-v | HPV 1-v | VIA 1-v | HPV 2-v | HPV 1-v | VIA 1-v | HPV 2-v | HPV 1-v | VIA 1-v | HPV 2-v | HPV 1-v |
| 20-24 | 0.0 | 0.0 | 0.0 | 0.0 | 0.0 | 0.0 | 0.0 | 0.0 | 0.0 | 0.0 | 0.0 | 0.0 | 0.0 | 0.0 | 0.0 |
| 25-29 | 0.0 | 0.0 | 0.0 | 0.0 | 0.0 | 0.0 | 0.0 | 0.0 | 0.0 | 0.0 | 0.0 | 0.0 | 0.0 | 0.0 | 0.0 |
| 30-34 | 1.5 | 3.4 | 4.6 | 0.0^b^ | 0.6 | 1.3 | 0.0^b^ | 0.0^b^ | 0.0^b^ | 0.0^b^ | 0.0^b^ | 0.0^b^ | 0.0^b^ | 0.0^b^ | 0.0^b^ |
| 35-39 | 7.0 | 8.9 | 11.1 | 7.6 | 8.7 | 11.1 | 8.1 | 8.9 | 11.3 | 7.9 | 9.0 | 11.2 | 7.7 | 9.1 | 11.1 |
| 40-44 | 5.9 | 7.3 | 9.3 | 6.1 | 7.5 | 9.6 | 6.6 | 7.9 | 10.1 | 6.7 | 8.7 | 11.1 | 6.7 | 8.7 | 11.0 |
| 45-49 | 4.6 | 5.8 | 7.8 | 4.8 | 6.6 | 8.5 | 4.9 | 6.9 | 8.8 | 5.3 | 7.6 | 9.7 | 5.6 | 7.7 | 9.9 |
| 50-54 | 3.4 | 5.3 | 6.5 | 3.5 | 5.4 | 6.9 | 3.6 | 5.7 | 7.1 | 3.9 | 6.1 | 7.6 | 4.8 | 6.3 | 7.9 |
| 55-59 | 3.0 | 4.5 | 5.6 | 3.2 | 4.9 | 6.0 | 3.5 | 4.9 | 6.1 | 3.6 | 5.1 | 6.3 | 3.8 | 5.3 | 6.6 |
| 60-64 | 2.5 | 4.1 | 5.0 | 2.6 | 4.3 | 5.3 | 2.7 | 4.6 | 5.6 | 2.7 | 4.9 | 6.0 | 2.9 | 4.9 | 6.1 |
| 65-69 | 1.6 | 3.0 | 3.8 | 1.7 | 3.3 | 4.0 | 1.7 | 3.8 | 4.6 | 2.0 | 3.9 | 4.9 | 2.0 | 4.0 | 5.1 |
| 70-74 | 1.3 | 2.1 | 2.5 | 1.4 | 2.3 | 2.6 | 1.3 | 2.4 | 2.7 | 1.4 | 2.5 | 2.9 | 1.6 | 2.7 | 3.2 |
| 75-79 | 0.4 | 1.4 | 1.6 | 0.5 | 1.6 | 1.9 | 0.6 | 1.8 | 2.0 | 0.6 | 1.8 | 2.2 | 0.7 | 2.2 | 2.6 |

^a^ HPV 1-v: 1-visit HPV DNA testing; HPV 2-v: 2-visit HPV DNA testing; VIA 1-v: 1-visit visual inspection with acetic acid. Reductions at age 75 to 79 years were also applied to women aged 80 to 100 years in CERVIVAC.

^b^ Where noted, cancer reductions estimated by the model were slightly negative, because an increase in the rate of detected cancers associated with screening outweighed the benefits of screening. In these instances, we assumed cancer reductions were zero to avoid biasing against screening.

**Table A.7. Age-specific cervical cancer mortality reduction (%) relative to no screening, by age at screening and screening strategy, at 10% coverage.**^a^

| Age  (years) | Screening at age 30 years | | | Screening at age 31 years | | | Screening at age 32 years | | | Screening at age 33 years | | | Screening at age 34 years | | |
| --- | --- | --- | --- | --- | --- | --- | --- | --- | --- | --- | --- | --- | --- | --- | --- |
|  | VIA 1-v | HPV 2-v | HPV 1-v | VIA 1-v | HPV 2-v | HPV 1-v | VIA 1-v | HPV 2-v | HPV 1-v | VIA 1-v | HPV 2-v | HPV 1-v | VIA 1-v | HPV 2-v | HPV 1-v |
| 20-24 | 0.0 | 0.0 | 0.0 | 0.0 | 0.0 | 0.0 | 0.0 | 0.0 | 0.0 | 0.0 | 0.0 | 0.0 | 0.0 | 0.0 | 0.0 |
| 25-29 | 0.0 | 0.0 | 0.0 | 0.0 | 0.0 | 0.0 | 0.0 | 0.0 | 0.0 | 0.0 | 0.0 | 0.0 | 0.0 | 0.0 | 0.0 |
| 30-34 | 5.4 | 3.6 | 4.4 | 2.5 | 1.5 | 1.6 | 1.9 | 1.8 | 2.1 | 0.3 | 0.3 | 0.3 | 0.3 | 0.0 | 0.0 |
| 35-39 | 2.2 | 3.8 | 4.6 | 2.1 | 4.2 | 4.7 | 2.4 | 3.5 | 4.0 | 2.3 | 3.5 | 3.8 | 1.5 | 2.5 | 2.7 |
| 40-44 | 2.1 | 3.1 | 3.9 | 1.9 | 2.7 | 3.7 | 2.0 | 2.6 | 3.4 | 2.3 | 2.9 | 3.8 | 2.0 | 2.6 | 3.5 |
| 45-49 | 3.1 | 3.5 | 4.3 | 3.1 | 3.7 | 4.4 | 3.5 | 3.6 | 4.3 | 3.2 | 4.2 | 4.9 | 3.2 | 4.1 | 4.6 |
| 50-54 | 1.8 | 2.7 | 3.5 | 1.9 | 2.9 | 3.8 | 1.9 | 2.8 | 3.6 | 2.2 | 3.2 | 4.1 | 2.2 | 3.1 | 4.0 |
| 55-59 | 1.7 | 2.7 | 3.4 | 1.8 | 2.8 | 3.5 | 1.9 | 3.0 | 3.8 | 2.0 | 2.8 | 3.7 | 2.1 | 3.0 | 3.9 |
| 60-64 | 1.5 | 2.5 | 3.2 | 1.6 | 2.8 | 3.5 | 1.7 | 2.5 | 3.3 | 1.8 | 2.7 | 3.4 | 1.9 | 2.8 | 3.5 |
| 65-69 | 1.5 | 2.4 | 3.0 | 1.4 | 2.3 | 2.9 | 1.5 | 2.4 | 3.0 | 1.5 | 2.4 | 3.1 | 1.6 | 2.7 | 3.5 |
| 70-74 | 1.0 | 1.5 | 1.9 | 0.9 | 1.6 | 2.0 | 1.0 | 1.8 | 2.3 | 0.9 | 1.7 | 2.3 | 1.1 | 1.8 | 2.4 |
| 75-79 | 0.7 | 1.2 | 1.5 | 0.9 | 1.5 | 1.8 | 0.7 | 1.4 | 1.6 | 0.8 | 1.6 | 1.8 | 0.8 | 1.4 | 1.7 |

| ^a^ HPV 1-v: 1-visit HPV DNA testing; HPV 2-v: 2-visit HPV DNA testing; VIA 1-v: 1-visit visual inspection with acetic acid. Reductions at age 75 to 79 years were also applied to women aged 80 to 100 years in CERVIVAC. |
| --- |
|  |
|  |

**Table A.8. Age-specific cervical cancer mortality reduction (%) relative to no screening, by age at screening and screening strategy, at 20% coverage.**^a^

| Age  (years) | Screening at age 30 years | | | Screening at age 31 years | | | Screening at age 32 years | | | Screening at age 33 years | | | Screening at age 34 years | | |
| --- | --- | --- | --- | --- | --- | --- | --- | --- | --- | --- | --- | --- | --- | --- | --- |
|  | VIA 1-v | HPV 2-v | HPV 1-v | VIA 1-v | HPV 2-v | HPV 1-v | VIA 1-v | HPV 2-v | HPV 1-v | VIA 1-v | HPV 2-v | HPV 1-v | VIA 1-v | HPV 2-v | HPV 1-v |
| 20-24 | 0.0 | 0.0 | 0.0 | 0.0 | 0.0 | 0.0 | 0.0 | 0.0 | 0.0 | 0.0 | 0.0 | 0.0 | 0.0 | 0.0 | 0.0 |
| 25-29 | 0.0 | 0.0 | 0.0 | 0.0 | 0.0 | 0.0 | 0.0 | 0.0 | 0.0 | 0.0 | 0.0 | 0.0 | 0.0 | 0.0 | 0.0 |
| 30-34 | 7.6 | 5.7 | 6.9 | 2.9 | 2.2 | 2.3 | 2.1 | 1.9 | 1.8 | 0.0^b^ | 0.0^b^ | 0.0^b^ | 0.2 | 0.0 | 0.0^b^ |
| 35-39 | 4.4 | 6.9 | 8.6 | 4.6 | 7.5 | 8.7 | 4.1 | 5.9 | 6.9 | 3.7 | 5.2 | 6.1 | 3.2 | 4.4 | 4.8 |
| 40-44 | 5.0 | 7.0 | 9.1 | 5.0 | 6.5 | 8.9 | 5.1 | 6.2 | 8.2 | 5.2 | 6.0 | 8.0 | 4.9 | 5.9 | 7.7 |
| 45-49 | 5.7 | 6.5 | 8.4 | 5.9 | 7.3 | 9.0 | 6.3 | 7.3 | 9.2 | 6.1 | 8.2 | 9.9 | 5.8 | 7.9 | 9.5 |
| 50-54 | 4.0 | 5.5 | 7.2 | 4.2 | 6.0 | 7.9 | 4.3 | 6.3 | 8.1 | 4.9 | 7.3 | 9.1 | 4.1 | 7.1 | 9.0 |
| 55-59 | 3.2 | 5.2 | 6.6 | 3.5 | 5.5 | 6.8 | 3.7 | 5.8 | 7.2 | 3.8 | 5.6 | 7.0 | 3.8 | 5.7 | 7.2 |
| 60-64 | 3.3 | 5.0 | 6.4 | 3.4 | 5.3 | 6.8 | 3.7 | 5.4 | 6.9 | 3.8 | 5.8 | 7.2 | 3.9 | 5.9 | 7.4 |
| 65-69 | 2.6 | 4.3 | 5.2 | 2.6 | 4.2 | 5.1 | 2.6 | 4.3 | 5.4 | 2.7 | 4.6 | 5.8 | 2.8 | 4.7 | 6.1 |
| 70-74 | 2.0 | 2.8 | 3.7 | 2.0 | 3.0 | 3.8 | 2.0 | 3.4 | 4.2 | 2.1 | 3.6 | 4.6 | 2.2 | 3.6 | 4.6 |
| 75-79 | 1.5 | 2.5 | 2.9 | 1.7 | 2.8 | 3.2 | 1.5 | 2.8 | 3.2 | 1.6 | 3.0 | 3.6 | 1.7 | 3.0 | 3.6 |

^a^ HPV 1-v: 1-visit HPV DNA testing; HPV 2-v: 2-visit HPV DNA testing; VIA 1-v: 1-visit visual inspection with acetic acid. Reductions at age 75 to 79 years were also applied to women aged 80 to 100 years in CERVIVAC.

^b^ Where noted, cancer reductions estimated by the model were slightly negative, because an increase in the rate of detected cancers associated with screening outweighed the benefits of screening. In these instances, we assumed cancer reductions were zero to avoid biasing against screening.

**Figure A1. Prevalence of high-risk HPV, India.** Selected model output from the top 50 input parameter sets compared with empirical data (i.e., calibration targets) on age-specific prevalence of high-risk HPV in India (Hyderabad), based on a relative light unit cut-off value of 0.5 in the START-UP studies.[^13^](#_ENREF_13) Bold lines represent the 95% confidence intervals around the empirical data, and gray circles represent model output from each of the top 50 input parameter sets.

**Figure A2. Cervical cancer incidence, India.** Selected model output from the top 50 input parameter sets compared with empirical data (i.e., calibration targets) on age-specific cancer incidence in India (Nagpur registry, 1998-2002).[^11^](#_ENREF_11) Bold lines represent the 95% confidence intervals around the empirical data, and gray circles represent model output from each of the top 50 input parameter sets.

**References**

1. Campos NG, Burger EA, Sy S, Sharma M, Schiffman M, Rodriguez AC, et al. An updated natural history model of cervical cancer: Derivation of model parameters. *Am J Epidemiol* 2014; **180**: 545-55.

2. Campos NG, Tsu, V., Jeronimo, J., Mvundura, M., Lee, K., Kim, J.J. When and how often to screen for cervical cancer in three low- and middle-income countries: A cost-effectiveness analysis. *Papillomavirus Research* 2015; **1**: 38-58.

3. Munoz N, Mendez F, Posso H, Molano M, van den Brule AJ, Ronderos M, et al. Incidence, duration, and determinants of cervical human papillomavirus infection in a cohort of colombian women with normal cytological results. *J Infect Dis* 2004; **190**: 2077-87.

4. Herrero R, Hildesheim A, Rodriguez AC, Wacholder S, Bratti C, Solomon D, et al. Rationale and design of a community-based double-blind randomized clinical trial of an hpv 16 and 18 vaccine in guanacaste, costa rica. *Vaccine* 2008; **26**: 4795-808.

5. McCredie MR, Sharples KJ, Paul C, Baranyai J, Medley G, Jones RW, et al. Natural history of cervical neoplasia and risk of invasive cancer in women with cervical intraepithelial neoplasia 3: A retrospective cohort study. *Lancet Oncol* 2008; **9**: 425-34.

6. Meyskens FL, Jr., Surwit E, Moon TE, Childers JM, Davis JR, Dorr RT, et al. Enhancement of regression of cervical intraepithelial neoplasia ii (moderate dysplasia) with topically applied all-trans-retinoic acid: A randomized trial. *J Natl Cancer Inst* 1994; **86**: 539-43.

7. Keefe KA, Schell MJ, Brewer C, McHale M, Brewster W, Chapman JA, et al. A randomized, double blind, phase iii trial using oral beta-carotene supplementation for women with high-grade cervical intraepithelial neoplasia. *Cancer Epidemiol Biomarkers Prev* 2001; **10**: 1029-35.

8. Castle PE, Schiffman M, Wheeler CM, Solomon D. Evidence for frequent regression of cervical intraepithelial neoplasia-grade 2. *Obstet Gynecol* 2009; **113**: 18-25.

9. Wang SM, Colombara D, Shi JF, Zhao FH, Li J, Chen F, et al. Six-year regression and progression of cervical lesions of different human papillomavirus viral loads in varied histological diagnoses. *Int J Gynecol Cancer* 2013; **23**: 716-23.

10. Moscicki AB, Ma Y, Wibbelsman C, Darragh TM, Powers A, Farhat S, et al. Rate of and risks for regression of cervical intraepithelial neoplasia 2 in adolescents and young women. *Obstet Gynecol* 2010; **116**: 1373-80.

11. Cancer incidence in five continents, vol. X [Internet]. IARC. 2013. Available from: <http://ci5.iarc.fr>.

12. Sankaranarayanan R, Nene BM, Shastri SS, Jayant K, Muwonge R, Budukh AM, et al. Hpv screening for cervical cancer in rural india. *N Engl J Med* 2009; **360**: 1385-94.

13. Jeronimo J, Bansil P, Lim J, Peck R, Paul P, Amador JJ, et al. A multicountry evaluation of carehpv testing, visual inspection with acetic acid, and papanicolaou testing for the detection of cervical cancer. *Int J Gynecol Cancer* 2014; **24**: 576-85.

14. Sankaranarayanan R, Esmy PO, Rajkumar R, Muwonge R, Swaminathan R, Shanthakumari S, et al. Effect of visual screening on cervical cancer incidence and mortality in tamil nadu, india: A cluster-randomised trial. *Lancet* 2007; **370**: 398-406.
